# Variability and pathogenicity of hepatitis E virus genotype 3 variants

**DOI:** 10.1099/jgv.0.000264

**Published:** 2015-11

**Authors:** Donald B. Smith, Samreen Ijaz, Richard S. Tedder, Boris Hogema, Hans L. Zaaijer, Jacques Izopet, Amanda Bradley-Stewart, Rory Gunson, Heli Harvala, Inka Kokki, Peter Simmonds

**Affiliations:** ^1^​ University of Edinburgh, CIIE, Ashworth Laboratories, King's Buildings, Edinburgh EH9 3FL, UK; ^2^​ Blood Borne Virus Unit, Virus Reference Department, MS-Colindale, Public Health England, London NW9 5EQ, UK; ^3^​ University College London, Gower Street, London WC1E 6BT, UK; ^4^​ Department of Blood-borne Infections, Sanquin Research, PO Box 9190, 1006 AD Amsterdam, The Netherlands; ^5^​ Institut National de la Sante et de la Recherche Medicale Unite 1043, Toulouse, France; ^6^​ West of Scotland Specialist Virology Centre, New Lister Building, Glasgow, UK; ^7^​ Specialist Virology Centre, Royal Infirmary of Edinburgh, UK; ^8^​ Public Health Agency of Sweden (previously Swedish Institute for Communicable Disease Control), Solna, Sweden; ^9^​ European Programme for Public Health Microbiology Training, European Centre for Disease Prevention and Control, Stockholm, Sweden; ^10^​ University of Edinburgh, Roslin Institute, Easter Bush, Edinburgh EH25 9RG, UK

## Abstract

Infection with hepatitis E virus (HEV) can be clinically inapparent or produce symptoms and signs of hepatitis of varying severity and occasional fatality. This variability in clinical outcomes may reflect differences in host susceptibility or the presence of virally encoded determinants of pathogenicity. Analysis of complete genome sequences supports the division of HEV genotype 3 (HEV-3) variants into three major clades: 3ra comprising HEV isolates from rabbits, and 3efg and 3abchij comprising the corresponding named subtypes derived from humans and pigs. Using this framework, we investigated associations between viral genetic variability of HEV-3 in symptomatic and asymptomatic infections by comparing HEV-3 subgenomic sequences previously obtained from blood donors with those from patients presenting with hepatitis in the UK (54 blood donors, 148 hepatitis patients), the Netherlands (38 blood donors, 119 hepatitis patients), France (24 blood donors, 55 hepatitis patients) and Germany (14 blood donors, 36 hepatitis patients). In none of these countries was evidence found for a significant association between virus variants and patient group (*P*>0.05 Fisher's exact test). Furthermore, within a group of 123 patients in Scotland with clinically apparent HEV infections, we found no evidence for an association between variants of HEV-3 and disease severity or alanine aminotransferase level. The lack of detectable virally encoded determinants of disease outcomes in HEV-3 infection implies a more important role for host factors in its clinical phenotype.

## Introduction

Infection with hepatitis E virus (HEV) is clinically silent in most individuals. For example, serological evidence of HEV infection is present in 13 % of the population of England and Wales (Ijaz *et al.*, 2009), equivalent to 100 000 infections annually (Hewitt *et al.*, 2014). However, the number of acute HEV infections reported annually in this population is currently < 1000, implying that 99 % of primary infections remain undiagnosed and are not associated with overt or currently recognized signs of disease.

One explanation for the low frequency of symptomatic infection with HEV is that different variants of HEV have different pathogenic potential. At present, four different genotypes of HEV have been described that infect humans; HEV-1 and HEV-2 are associated with endemic transmission in developing countries, whilst HEV-3 and HEV-4 appear to be zoonotic, resulting from the consumption of undercooked pig meat in developing countries (Smith *et al.*, 2014). Whilst a high fatality rate has been reported for pregnant women infected with HEV-1 (Patra *et al.*, 2007), this has not been reported for HEV-3 or HEV-4. Different pathogenic associations have also been described for HEV-3 and HEV-4 (Abe *et al.*, 2006; Jeblaoui *et al.*, 2013; Mizuo *et al.*, 2005; Ohnishi *et al.*, 2006), as well as for particular variants of HEV-3 (Takahashi *et al.*, 2009), whilst the development of fulminant hepatitis has been related to the presence of particular nucleotide substitutions in the genomes of HEV-1 and HEV-3 (Bu *et al.*, 2013; Inoue *et al.*, 2006, 2009; Pujhari *et al.*, 2010; Sugawara *et al.*, 2009).

Alternatively, some of these differences in the outcome of HEV infection might result from variable host responses and disease susceptibility, such as the extent of pre-existing liver damage or the presence of particular immunological reactivities. Such differing susceptibilities, and possibly an age-cohort effect, might explain the skewed distribution of HEV-3 infection in European countries, with most patients being >50 years of age, male and with a history of pre-existing liver disease or excessive alcohol use.

We attempted to discriminate between these two possibilities for HEV-3 by comparing the distribution of variants observed in blood donors and hepatitis patients using previously published data from several European countries. By definition, HEV-infected hepatitis patients have raised liver function tests and/or jaundice, often with an additional wide spectrum of presenting symptoms, including vomiting, nausea, dark urea, malaise, abdominal pain, lethargy and anorexia/loss of appetite. A small proportion of patients included in this group are immunosuppressed with chronic HEV-3 infection, but without symptoms of hepatitis. In contrast, the majority of blood donors are immunocompetent and display no or only mild symptoms of hepatitis, at least at the time of donation. Although liver function tests are no longer used as a marker to exclude donations from individuals with viral hepatitis, retrospective testing shows that most HEV-3-infected blood donors have normal or only slightly elevated liver function tests (Juhl *et al.*, 2014; Vollmer *et al.*, 2012). If different variants were observed in these two patient groups, this would provide evidence that virus variation was involved in differences in disease outcome.

A complication to undertaking this analysis was the lack of consensus on how variants of HEV-3 should be classified. An earlier study identified 10 different virus subtypes (3a–3j) on the basis of comparisons of complete genome sequences or of different subgenomic regions (Lu *et al.*, 2006). However, as more virus sequences have become available the distribution of sequence distances within HEV-3 has become more continuous so that no consistent criteria have been identified that distinguish between variants that belong to the same or different subtypes (Oliveira-Filho *et al.*, 2013; Smith *et al.*, 2013a, 2014). In addition, several recent studies have identified higher-level groupings, variously described as subgroups 3.1 and 3.2 (Oliveira-Filho *et al.*, 2013), groups 3-I and 3-II (Bouquet *et al.*, 2012; Widén *et al.*, 2011), groups 1 and 2 (Hewitt *et al.*, 2014; Ijaz *et al.*, 2014) or groups 3jab, 3chi and 3feg (Vina-Rodriguez *et al.*, 2015). However, these various groupings of variants utilize different reference sequences and include different sets of named subtypes.

Here, we describe a reanalysis of the extent of variation and nomenclature of HEV-3 subtypes and higher-level groupings. We then used a reference set of sequences of named subtypes in order to answer the question of whether or not specific variants of HEV-3 were responsible for different disease outcomes. We compared published data for HEV-3 sequences obtained from European blood donors and hepatitis patients as well as from variants infecting individuals with differing severity of hepatitis. In neither case did we find evidence that virus variation determined pathogenicity.

## Results

### Variation within HEV-3

**Fig. 1.**
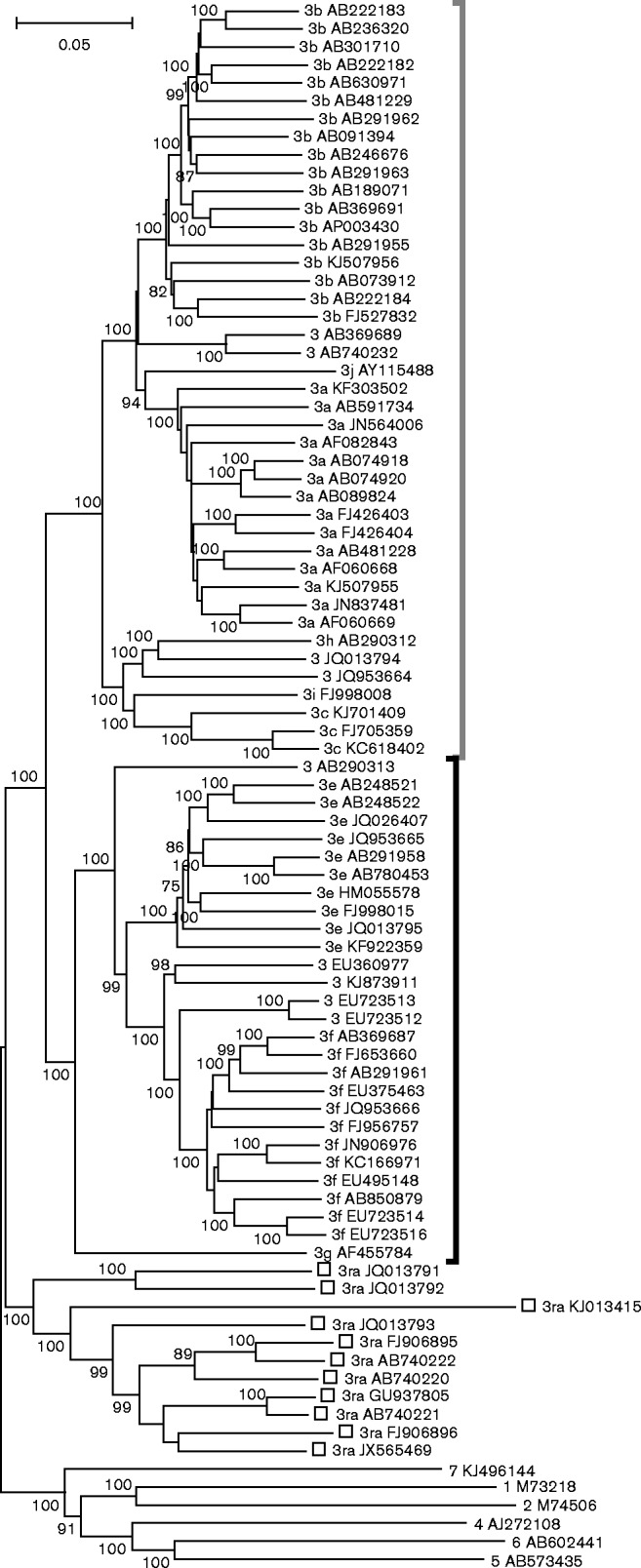
Phylogenetic analysis of HEV-3 complete genome sequences (GenBank accession numbers). A neighbour-joining tree of 81 HEV-3 complete genome sequences together with reference sequences of other genotypes was produced using maximum composite likelihood distances. Named subtypes of 3abchij are enclosed by a grey bracket and those of 3efg by a dark bracket. Isolates belonging to clade 3ra are identified by open squares. Bootstrap support (>70 %) is indicated for individual branches.

Variants of HEV-3 differ by up to 26 % of nucleotides over their complete genome sequences, with differences of up to 22, 12 and 22 % in ORF1, ORF2 and ORF3 amino acid sequence, respectively. Analysis of 81 complete, non-redundant HEV-3 coding sequences together with reference sequences of the other known genotypes revealed three major groups (Fig. 1). Similar relationships were observed if the hypervariable regions were omitted, if analysis was conducted by the maximum-likelihood method or if concatenated ORF1/ORF2 amino acid sequences were used, although in the latter case branches separating different groups were much shallower with lower percentage bootstrap support (data not shown).

One well-defined, but diverse, group of strains (3ra) includes sequences first isolated from rabbits and currently represented by a single human isolate (GenBank accession number JQ013793); all of these sequences contain a distinctive 93 hypervariable nucleotide insertion within ORF1 and downstream of the hypervariable region (HVR). Although we treated these isolates here as divergent members of HEV-3 (Smith *et al.*, 2013a, 2014), other publications separated them into a novel genotype (Geng *et al.*, 2011; Izopet *et al.*, 2012; Zhao *et al.*, 2009). The remaining variants of HEV-3 can be consistently divided into two major clades: 3abchij (including subtypes 3a, 3b, 3c, 3h, 3i, 3j and four unclassified variants) and 3efg (including subtypes 3e, 3f, 3g and five unclassified variants). These two clades correspond to the previously described groups 3.1 and 3.2 (Oliveira-Filho *et al.*, 2013), groups 3-I and 3-II (Widén *et al.*, 2011), and group 2 and group 1 (Hewitt *et al.*, 2014; Ijaz *et al.*, 2014).

**Fig. 2.**
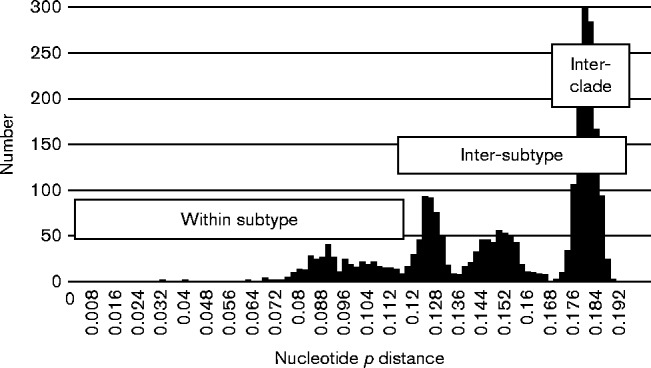
Frequency histogram of nucleotide *p* distances amongst HEV-3 variants. Pairwise distances amongst the complete genome sequences (from which the HVR had been removed) of the HEV-3 sequences shown in Fig. 1, but excluding the 3ra variants. The distributions of sequence distances within subtype, between subtypes and between clades (3abchij and 3efg) are indicated.

Pairwise nucleotide distances (excluding the HVR) amongst and between the 3abchij and 3efg clades comprise an almost continuous distribution that has several distinct peaks (Fig. 2). A peak centred on distances of 0.182 comprises comparisons between members of the two clades. Distances within individual subtypes 3a, 3b, 3c, 3e and 3f (as labelled on Fig. 1) include two major peaks and range up to 0.114, but these overlap the distribution of distances between different subtypes, the lowest inter-subtype distance being 0.113 between AB091394 (3b) and AB089824 (3a). In this context it is not clear how to assign isolates with distances to defined subtypes that span this region (distances to 3f sequences of 0.09–0.117 for EU723513 and EU723512 and 0.112–0.119 for EU360977). In the absence of consistent criteria for defining subtypes we have taken the decision not to assign these or other divergent isolates to the 10 existing subtypes (Lu *et al.*, 2006) or to novel subtypes. Our assignments are therefore more conservative than those of a recent publication (Vina-Rodriguez *et al.*, 2015) in which the unassigned variants were classified as 3f (EU723513, EU723512, EU360977 and KJ873911), 3h (JQ013794 and JQ953664) 3j (AB740232 and AB369689) or ‘3ef’ (AB290313).

The peak centred on 0.125 comprises distances within each of the three proposed subclade groupings of 3jab, 3chi and 3efg (Vina-Rodriguez *et al.*, 2015). However, the peak centred on 0.15 includes distances between and within these groupings, an overlap that is not resolved if 3g is treated as a fourth subclade group. This suggests that Figure 2 of Vina-Rodriguez *et al.* (2015) has been mislabelled and that the three proposed subgroupings are not defined by a discrete range of sequence distances.

Whilst conducting this analysis, we identified several conflicts between published subtype designations. Sequence comparisons with the subtype prototype sequences (Lu *et al.*, 2006) suggest that FJ705359 and KC618402, previously described as 3i (Hewitt *et al.*, 2014; Johne *et al.*, 2014), are subtype 3c; that JQ013794, previously described as 3c (Izopet *et al.*, 2012), is an unassigned variant; AB290312 is 3h; EU360977, AB248522 and AB248521, all previously described as 3c (Xia *et al.*, 2008), are an unassigned variant, 3e and 3e, respectively; and AB740232 and AB369689, previously described as 3j, (Vina-Rodriguez *et al.*, 2015) are unclassified variants. Although AY115488 was obtained from pooled material, we have retained it as 3j as we have found no evidence that it is a recombinant between known subtypes. We note that no complete genome sequence is currently available that corresponds to 3d of Lu *et al.* (2006).

### Pathogenicity of HEV-3 variants

Using this classification framework, we next investigated whether there was evidence for differences in pathogenicity between variants of HEV-3 by comparing their distribution in cases of hepatitis and blood donors. The rationale for this was that individuals infected with HEV-3 with a diagnosis of hepatitis must have developed one or more symptoms of hepatitis in order to have been considered for diagnostic testing. Such symptoms would include jaundice, abnormal liver function tests [alanine aminotransferase (ALT) level >100 U l^− 1^], abdominal pain or dark urine, but might also extend to more general symptoms, such as malaise, anorexia, fever or neurological involvement. In contrast, blood donations would not be accepted from individuals known to have any of these symptoms at the time of donation. Most HEV-3-infected blood donors have normal or slightly elevated ALT levels (Juhl *et al.*, 2014; Vollmer *et al.*, 2012) (H. Zaaijer, unpublished results). If there was a difference in the pathology of different HEV-3 variants, then it might be expected that the distribution of these variants would differ between these two groups of HEV-3-infected individuals with overt or silent HEV-3 infection.

**Fig. 3.**
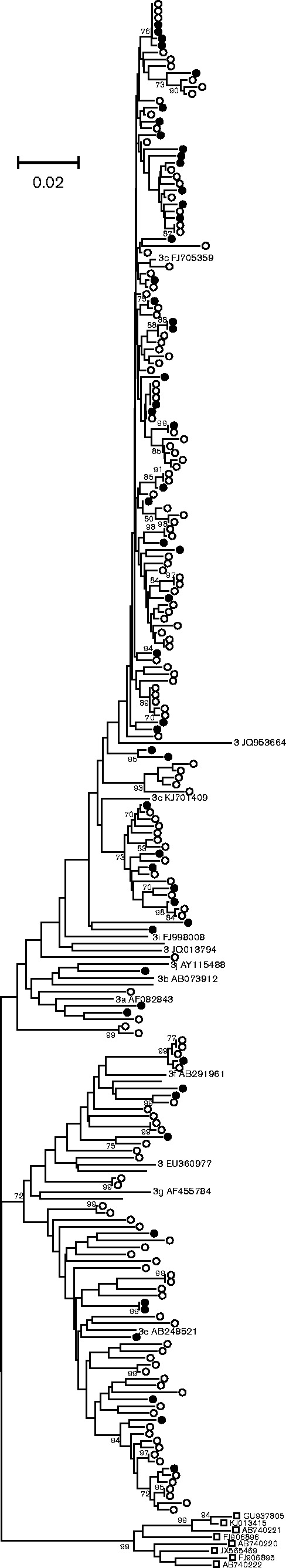
Phylogenetic analysis of HEV-3 variants from blood donors and hepatitis patients in England and Wales. HEV-3 ORF2 sequences (280 nt, nt 6041–6320 numbered relative to AF082843) isolated from blood donors (•) and hepatitis patients (○) were compared with reference sequences of named HEV-3 subtypes and 3ra isolates (▪).

For England and Wales, HEV-3 ORF2 sequences (280 nt) were obtained from 54 blood donors in 2012/2013 and 508 hepatitis patients spanning the period 2003–2012 (Hewitt *et al.*, 2014; Ijaz *et al.*, 2014). Comparison of these two sets of virus sequences was complicated by the observation that the distribution of variants detected in hepatitis patients changed over time with a shift from 3efg (group 1), which predominated before 2009, to 3abchij (group 2), which became the dominant variant after 2011 (Ijaz *et al.*, 2014) and in 2013 comprised 69 % of isolates (S. Ijaz, unpublished results). Considering only the 148 HEV-3 sequences obtained during 2012 and comparing these with the 54 blood donor-derived HEV-3 sequences detected in 2012/2013, it is apparent that variants from both patient groups were distributed widely within the HEV-3 phylogeny (Fig. 3). In particular, 11 of the 62 clade 3efg sequences were from blood donors (18 %), whilst their proportion within clade 3abchij was 43 of 140 (30 %), a distribution that was not significantly different by Fisher's exact test (*P* = 0.06). An association index (AI) value of 0.85 similarly provided no evidence for a difference in clustering of HEV-3 variants from blood donors and hepatitis patients into phylogenetically distinct clades or subtypes.

**Fig. 4.**
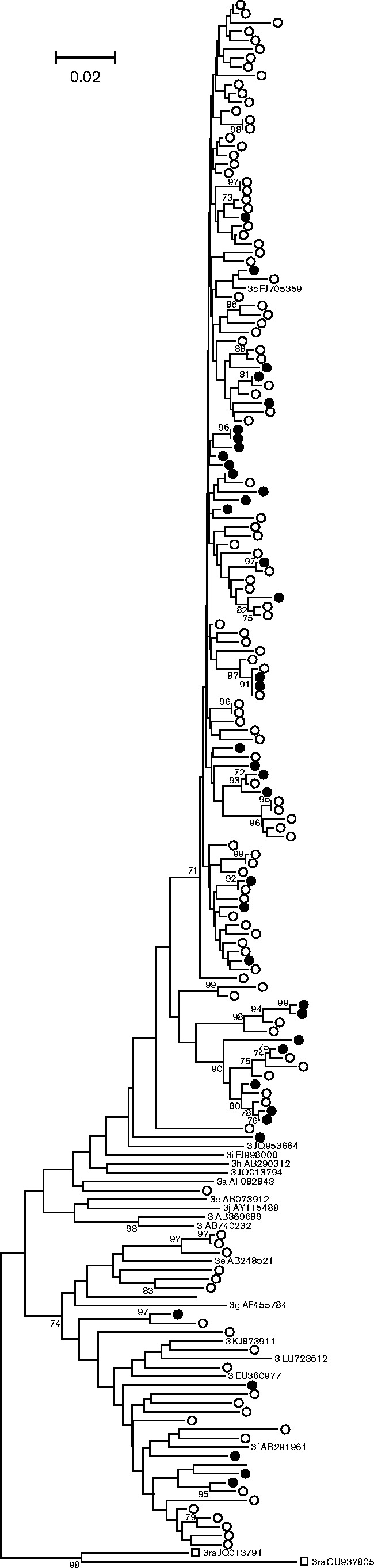
Phylogenetic analysis of HEV-3 variants from blood donors and hepatitis patients in the Netherlands. ORF2 sequences (304 nt, nt 6018–6321) isolated from blood donors (•) and hepatitis patients (○) were compared with reference sequences of named HEV-3 subtypes and 3ra isolates (▪).

Similar comparisons were made for a dataset from the Netherlands including ORF2 sequences (304 nt) from 38 blood donors (2011–2014) and 119 hepatitis patients (2010–2014) (Fig. 4). In contrast to England and Wales, no change in the distribution of HEV-3 variants was apparent in a study of 34 Dutch patients with unexplained hepatitis over the period 2007–2012 (Riezebos-Brilman *et al.*, 2013). Of the 28 clade 3efg sequences, five were derived from blood donors (18 %) compared with 33 of 123 clade 3abchij sequences (27 %), a distribution that was not significantly different by Fisher's exact test (*P* = 0.47) or the AI test (0.93). Similar results were obtained from the analysis of ORF1 sequences from the same patients or from concatenated ORF1/ORF2 sequences (results not shown).

**Fig. 5.**
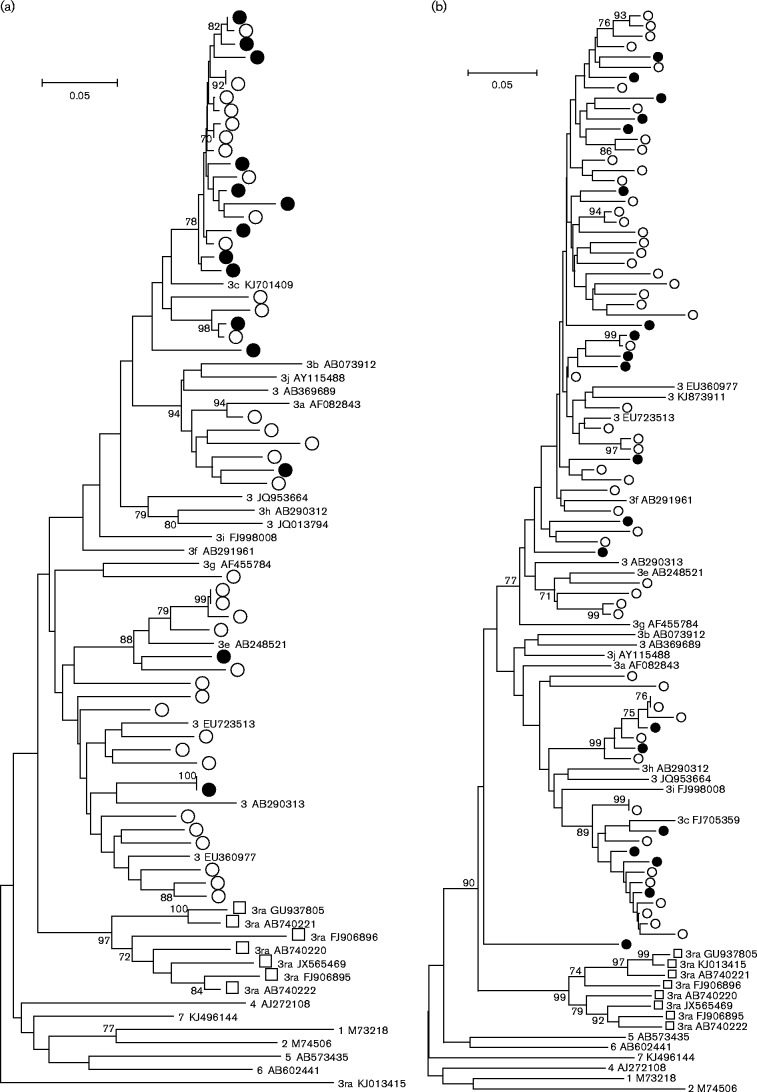
Phylogenetic analysis of HEV-3 variants from blood donor and hepatitis patients in (a) Germany (ORF1 nt 107–348) and (b) France (ORF2 nt 5996–6342): blood donors (•) and hepatitis patients (○) together with named subtypes and 3ra isolates (▪). Branches with >70 % bootstrap support are indicated.

Smaller datasets are available from France and Germany, but in these cases it was not possible to match the year of sampling for hepatitis and blood donor groups. Sequences of two different genomic regions were obtained for HEV-3 derived from German blood donors. Analysis of 14 ORF1 sequences (242 nt) from blood donors (Huzly *et al.*, 2014; Vollmer *et al.*, 2012) and 36 hepatitis patients revealed that two blood donors and 18 hepatitis patients grouped with 3efg, whilst 12 blood donors and 18 hepatitis patients grouped with 3abchij (Fig. 5a), a distribution that was not significantly different by Fisher's exact test (*P* = 0.06) or the AI test (0.92). Analysis of a different region of ORF1 in an additional 15 blood donors (Corman *et al.*, 2013; Drexler *et al.*, 2012; Huzly *et al.*, 2014) again revealed a bias towards 3abchij (five in clade 3efg, 10 in clade 3abchij), but no equivalent sequences for this genome region were available for German hepatitis patients. Finally, analysis of 24 HEV sequences derived from French blood donors (Gallian *et al.*, 2014) and 55 French hepatitis patients from 2007 to 2010 (Fig. 5b) revealed that 13 blood donor and 40 hepatitis patient sequences clustered with 3efg, and six blood donor and 14 hepatitis patients clustered with 3abchij, a distribution that was not significantly different by Fisher's exact test (*P* = 0.77) or the AI test (0.77). However, we note that the distribution of French blood donor sequences amongst clades 3efg and 3abchij (13 and six) does differ from that observed in England and Wales (11 and 43, *P* = 0.0004 Fisher's exact test) or amongst European blood donors (23 and 98, *P* < 0.0001). The distribution was also significantly different for hepatitis patients from France (40 3efg, 14 3abchij) and England and Wales (51 3efg, 97 3abchij, *P* < 0.0001) or Europe (92 3efg, 205 3abchij, *P* < 0.0001).

We also investigated the pathogenicity of different HEV-3 variants by measuring their association with the symptoms reported in HEV-3-infected patients. Nucleotide sequences in the ORF2 region (nt 6058–6320) were obtained for 123 HEV-3-infected individuals in Scotland over the period 2012–2015 (Edinburgh *n* = 25, Glasgow *n* = 74) and collated with clinical information. Presenting clinical symptoms (apart from ALT levels, which were >100 U l^− 1^ for all patients) were classed as non-specific (malaise, nausea), overt (jaundice, dark urine, abdominal pain, fever) or unknown (Table 1). ALT levels at the time of referral were categorized as < 2000 or ≥ 2000 U l^− 1^. Neither the severity of symptoms nor ALT levels were significantly associated with clades 3efg/3abchij, or with subclades 3e/3f or 3c/3abhij by Fisher's exact test (*P* = 1 and 0.76, respectively) or the AI test (*P* = 0.98 and 1.04, respectively).

**Table 1.** Clinical correlates with virus groupings amongst HEV-3 patients in Scotland

Clinical data	Total (*n* = 123)	3abchij (*n* = 95)	3efg (*n* = 28)	3abhij (*n* = 7)	3c (*n* = 88)	3e (*n* = 20)	3f (*n* = 8)
**ALT level** **(U l** ^** − 1**^ **)**							
Unknown	33	28	5	2	26	4	1
< 2000	58	45	13	3	42	8	5
≥ 2000	32	22	10	2	20	8	2
**Symptoms**							
Unknown	19	13	6	1	12	4	2
Non-specific	56	47	9	1	46	7	2
Overt	48	35	13	5	30	9	4
**Combined**							
Unknown	39	31	8	2	29	6	2
ALT < 2000 U l^− 1^ or non-specific	63	51	12	3	48	8	4
ALT ≥ 2000 U l^− 1^ and overt	21	13	8	2	11	6	2

## Discussion

HEV-3 displays considerable diversity and various authors have proposed, sometimes contradictory, classification schemes that assign these to different subtypes (Lu *et al.*, 2006; Xia *et al.*, 2008) or groups (Ijaz *et al.*, 2014; Oliveira-Filho *et al.*, 2013). Phylogenetic analysis of HEV-3 complete genome sequences suggests an initial division of HEV-3 into three clades (Fig. 1) comprising 3ra, variants first isolated from rabbits, and clades 3efg and 3abchij that derive from humans, pigs, wild boar or deer. However, further division of these clades into subgroupings or subtypes becomes arbitrary as pairwise distances between sequences form continuous distributions (Fig. 2) with multiple subsidiary branches supported by bootstrap replication (Fig. 1).

Using this framework, we have undertaken a comparison of the pathogenicity of different variants of HEV-3 by comparing their distribution in blood donors and patients with hepatitis. Analysis of nucleotide sequences from the UK (Fig. 3), the Netherlands (Fig. 4), Germany (Fig. 5a) and France (Fig. 5b) reveals that the distribution of viruses from clades 3efg and 3abchij is similar between these two patient groups. This is consistent with the idea that HEV-3 infection is clinically silent in the majority of individuals and that the rare cases in which hepatitis or other overt pathologies are observed reflect differences in the host susceptibility or response to infection rather than differences in the pathogenesis of different virus variants. We also observed no difference in the distribution of HEV-3 variants between hepatitis patients from Scotland with symptoms of hepatitis compared with those without such symptoms or to those with ALT levels < 2000 or ≥ 2000 U l^− 1^ (Table 1).

The only amino acid polymorphism that differed between blood donors and hepatitis patients in the sequence sets we compared was a Leu → Phe substitution in ORF2 (nt 6002 numbered with reference to GenBank accession number AF082843). This substitution was present in 14 of 20 French blood donor sequences (another three had mixed sequences at this position) compared with none of 23 amongst French hepatitis patients. This substitution was also present in four of 48 UK blood donor sequences, but was not sequenced for the corresponding hepatitis patients, or any of the sequences from the Netherlands or Germany.

Our analysis is unable to exclude the possibility that differences in pathogenicity are due to substitutions occurring in parts of the virus genome outside the subgenomic regions considered here (methyltransferase, RNA-dependent RNA polymerase and ORF2). However, such changes would have to segregate independently of the (largely) synonymous substitutions upon which our phylogenetic analyses are based. For example, pathogenic substitutions might arise at a high frequency and so segregate independently of phylogeny. Several previous studies found evidence that particular nucleotide substitutions in HEV-1, HEV-3 and HEV-4 were associated with severe pathology, such as severe acute hepatitis or fulminant hepatitis (Bu *et al.*, 2013; Inoue *et al.*, 2006, 2009; Pujhari *et al.*, 2010; Sugawara *et al.*, 2009; Takahashi *et al.*, 2009). However, these associations may arise because of the phylogenetic linkage between viruses sampled from restricted geographical regions rather than as determinants of pathogenicity (Smith & Simmonds, 2015).

Another potential scenario is that pathogenic substitutions arise rarely and move between lineages through the process of inter-lineage recombination. However, there are very few reports of infection with multiple lineages (Moal *et al.*, 2012; Smith *et al.*, 2013b) and recombination in HEV is infrequent (Chen *et al.*, 2012; Fan, 2009; van Cuyck *et al.*, 2005; Wang *et al.*, 2010), and in some cases artefactual, resulting from mixed infection or laboratory contamination (Wang *et al.*, 2010). The identification of lineage-independent pathogenic substitutions would require a major effort to obtain complete genome sequences from large numbers of HEV-3-infected individuals from a limited geographical region, but with divergent disease outcomes. Differences in virus pathogenicity might also be affected by the virus titre at the time of infection; our analysis could not detect such an association unless the infecting titre was correlated with specific subtypes.

In conclusion, we find no evidence to support the hypothesis that the pathogenesis of HEV-3 infection is virus dependent. Our previous analysis of the relationship between HEV infection and the development of fulminant hepatitis reached a similar conclusion in respect of this severe and unusual outcome of infection (Smith & Simmonds, 2015). Exposure to HEV-3 infection appears to be general and cumulative, with severe disease restricted to a subset of individuals, particularly older men with a history of liver disease or excessive alcohol consumption, or amongst individuals with reduced immune function.

## Methods

### Nucleotide sequences

Complete genome sequences (accession numbers) were obtained from GenBank on 2 February 2015 and comprised single representatives of each genotype together with all non-redundant HEV-3 sequences (i.e. that differed from each other by ≥ 2 % of nucleotide positions, not including the HVR, so as to prevent multiple sampling of similar sequences from biasing the analyses): 1 M73218, 2 M74506, 3a AF082843, 3a AB074918, 3a AB074920, 3a AB089824, 3a AB481228, 3a AB591734, 3a FJ426403, 3a FJ426404, 3a JN837481, 3a KF303502, 3a KJ507955, 3a AF060669, 3a AF060668, 3a JN564006, 3b AB073912, 3b AB091394, 3b AB189071, 3b AB222182, 3b AB222183, 3b AB222184, 3b AB236320, 3b AB246676, 3b AB291955, 3b AB291962, 3b AB291963, 3b AB301710, 3b AB369691, 3b AB481229, 3b AP003430, 3b FJ527832, 3b KJ507956, 3b AB630971, 3c FJ705359, 3c KC618402, 3c KJ701409, 3e AB248521, 3e AB248522, 3e AB291958, 3e AB780453, 3e HM055578, 3e JQ953665, 3e KF922359, 3e JQ026407, 3e JQ013795, 3e FJ998015, 3 EU360977, 3f AB291961, 3f AB369687, 3f EU375463, 3f EU495148, 3f EU723514, 3f EU723516, 3f FJ653660, 3f FJ956757, 3f JN906976, 3f JQ953666, 3f KC166971, 3f AB850879, 3 EU723513, 3 EU723512, 3 KJ873911, 3g AF455784, 3h AB290312, 3 JQ013794, 3i FJ998008, 3j AY115488, 3 JQ953664, 3 AB290313, 3 AB369689, 3 AB740232, 3ra JQ013791, 3ra JQ013792, 3ra JQ013793, 3ra GU937805, 3ra KJ013415, 3ra FJ906896, 3ra FJ906895, 3ra AB740222, 3ra AB740221, 3ra AB740220, 3ra JX565469, 4 AJ272108, 6 AB602441, 5 AB573435 and 7 KJ496144.

HEV-3 sequence datasets (GenBank accession numbers) from blood donors and hepatitis patients were as follows. (1) England and Wales – blood donors: KT004596–KT004649; England and Wales patients 2011/12: KF513874, KF513881, KF513884–KF513890, KF513892–KF513901, KF513903–KF513912, KF513914–KF513923, KF513926–KF513935, KF513937–KF513946, KF513948–KF513957, KF513959–KF513968, KF513970–KF513980, KF513981–KF513990, KF513992–KF514001, KF514003–KF514012, KF514014–KF514023, KF514025–KF514034 and KF514037–KF514045. (2) The Netherlands – blood donors: JX645320–JX645333, KR362770–KR362792 and JX678984. (3) The Netherlands – hepatitis patients: JX645334–JX645340, KM820620–KM820660, KR362793–KR362858, JQ929119–JQ929124 and KC758127–KC758130. (4) Germany – blood donors (RNA-dependent RNA polymerase): JQ034513, JX110665, JQ034518, JQ034517, KJ873912, KJ873911, JX110666, FJ956757, JQ034512, JQ034521, JQ034520, JQ034519, JQ034514, JQ034515, JQ034522, JQ034516 and JX110667. (5) Germany – blood donors (methyltransferase): JQ863414, JQ863408, JQ863412, JQ863409, JQ863407, JQ863416, JQ863406, JQ863418, JQ863417, JQ863415, JQ863413, JQ863411, JQ863410 and KJ547592. (6) Germany – hepatitis patients (methyltransferase): EU879113, FN985025, EU879099, FN994997, HE605115, EU879112, FR728253, EU879100, FR846451, HE605114, AJ889194, FN995000, EU879111, HF912156, EU879110, FR728248, FR846450, FR728255, FR728250, FN994999, HE605113, FN985026, EU879118, EU879109, GU479457, EU879117, FN994998, FR687017, EU879098, EU879106, FN985024, FR728251, EU879114, EU879116, EU879102 and EU879103. (7) France – blood donors (ORF2): KJ740414–KJ740435. (8) France – patients: JN906974, HQ682232, HQ688787, JQ763789, JQ763893, JQ763895, JQ763954, EU514957, EF050799, EU350572, EU369392, EU369391, EU543566, EU340256, GQ240310, EU236150, JQ013794, GQ427004, EU543564, EU543567, GU994211, KJ742843, JQ764029, JQ763703, JQ763611, GQ427014, EU650323, EU667419, KJ742844, KJ742845, GU936617, EU667421, EU650322, EU650321, EU532016, GQ426997, EU667420, EU667422, KF751184, GU084155, EU495148, EU650320, JQ763653, JQ763821, JQ763774, KJ742842, KJ701409, GQ426990, GQ426985, GQ427009, KJ742841, HM066937, KJ742846 and JQ763823.

### Virus sequence analysis

Virus nucleotide sequences from anonymized hepatitis patient samples were obtained from hepatitis patients referred to the Edinburgh Royal Infirmary as part of ongoing epidemiological studies of patients with raised ALT (>100 U l^− 1^) or with other signs of unexplained hepatitis as described previously (Ramalingam *et al.*, 2013). Virus nucleotide sequences from patients referred to the West of Scotland Specialist Virology Centre, Glasgow were obtained from anonymized samples by reverse transcription-PCR using the ORF2 primers 3156 [5′-AAT(C)TATGCC(A)CAGTACCGGGTTG-3′], 3157 (5′-CCCTTATCCTGCTGAGCATTCTC-3′), 3158 [5′-GTT(C)ATGC(T)TT(C)TGCATACATGGCT-3′] and 3159 [5′-AGCCGACGAAATC(T)AATTCTGTC-3′] using standard procedures. GenBank accession numbers of the sequence obtained here were KP835485–KP835511 (Edinburgh), and KR340493–KR340578 and KT119521–KT119531 (Glasgow).

### Phylogenetic and statistical analysis

Sequences were aligned and annotated using sse version 1.2 (Simmonds, 2012). Phylogenetic analysis was performed using mega6 (Tamura *et al.*, 2013). The significance of the distribution of HEV variants associated with blood donors or hepatitis patients, or amongst hepatitis patients with different symptoms of infection or ALT levels, was assessed using Fisher's exact test. AI calculations (Cochrane *et al.*, 2002) that linked virus clades to clinical outcome were carried out within sse, as were Bootscan and Groupscan analysis of AY115488.
